# Complete Genome Sequences of Two Strains of Xanthomonas campestris pv. campestris Isolated in Japan

**DOI:** 10.1128/MRA.01239-19

**Published:** 2020-01-09

**Authors:** Kasumi Takeuchi, Ichiro Mitsuhara

**Affiliations:** a Division of Plant and Microbial Sciences, Institute of Agrobiological Sciences, National Agriculture and Food Research Organization, Tsukuba, Ibaraki, Japan; Broad Institute

## Abstract

Here, we report the complete genome sequences of two strains of Xanthomonas campestris pv. campestris (MAFF106712 and MAFF302021), which cause black rot in crucifer crops, isolated from Chinese cabbage and cauliflower, respectively, in Japan. The MAFF106712 chromosome was 5,002,720 bp, with a G+C content of 65.2%, and harbored one plasmid of 78,747 bp. The MAFF302021 chromosome was 5,048,651 bp, with a G+C content of 65.1%.

## ANNOUNCEMENT

Xanthomonas campestris pv. campestris is a causal agent of black rot in crucifer crops. It causes disease in a large number of species of *Brassicaceae*, including economically important vegetable *Brassica* crops worldwide. Warm and wet conditions favor plant infection by X. campestris pv. campestris; however, practical approaches for the management of this pathogen are limited. To obtain a deeper understanding of this pathogen, we report here the complete genome sequences of two strains of X. campestris pv. campestris, MAFF106712 and MAFF302021, isolated in Japan. Their growth conditions have been described in detail (1). Strain MAFF106712 was isolated from the infected leaves of Chinese cabbage (Brassica rapa subsp. pekinensis) growing in a field in Ibaraki. Strain MAFF302021 was isolated from the infected leaves of cauliflower (Brassica oleracea) growing in a field in Shiga. Regarding the isolation of these genome sequences, the two strains were grown at 28°C overnight in 10 ml of nutrient yeast broth (NYB) (2.5% [wt/vol] nutrient broth and 0.5% [wt/vol] yeast extract) with shaking at 160 rpm. The genome sequences of MAFF106712 and MAFF302021, which were isolated using the CsCl density gradient centrifugation method (2), were sequenced as follows. The integrity of the genomic DNA was checked by agarose gel electrophoresis, and it was then quantified using the Quant-IT PicoGreen kit (Invitrogen). Sequencing libraries were prepared according to the manufacturer’s instructions with a 20-kb template preparation using the BluePippin size selection system. Briefly, 8 μg of genomic DNA was sheared to 20 kb using g-TUBES (Covaris), purified, and end repaired, and blunt-end SMRTbell adapters were then ligated. The libraries were quantified using Quant-IT PicoGreen (Invitrogen) and a 12000 DNA chip (Agilent Technologies, Waldbronn, Germany). Libraries were prepared for sequencing by annealing a sequencing primer (a component of the SMRTbell template prep kit 1.0) and binding polymerase to the primer-annealed the template. The polymerase-bound template was bound to MagBeads. The libraries were then sequenced using PacBio P6-C4 chemistry in 8-well single-molecule real-time (SMRT) cell v3 in the PacBio RS II platform (Macrogen, Inc., Japan) (3).

Genome libraries consisting of 96,180 and 97,524 reads with *N*_50_ values of 17,307 and 17,374 bp were obtained for MAFF106712 and MAFF302021, respectively. Genome *de novo* assembly was performed using Unicycler (version 0.4.6) (4), with default settings, resulting in two contigs corresponding to one chromosome and one plasmid in MAFF106712 and one contig corresponding to one chromosome in MAFF302021. The length of the whole-genome sequence obtained for MAFF106712 was 5,081,467 bp, consisting of one chromosome (5,002,720 bp) and one plasmid (78,747 bp), with average read depths of 137× for the chromosome and 204× for the plasmid. The length of the whole-genome sequence obtained for MAFF302021 was 5,048,651 bp, with an average read depth of 150×.

Genome annotations of X. campestris pv. campestris MAFF106712 and MAFF302021 were performed using the DDBJ Fast Annotation and Submission Tool (DFAST) pipeline (5). The annotated genome of MAFF106712 contained 4,422 coding DNA sequences (CDSs), 59 tRNA genes, and 6 rRNA genes. The annotated genome of MAFF302021 contained 4,421 CDSs, 60 tRNA genes, and 6 rRNA genes.

### Data availability.

The complete genome sequences have been deposited in DDBJ/GenBank under the accession numbers AP019682 and AP019683 for the MAFF106712 chromosome and plasmid, respectively, and AP019684 for the MAFF302021 chromosome. The BioProject accession number is PRJDB8187, and the BioSample accession numbers are SAMD00166934 and SAMD00166935 for MAFF106712 and MAFF302021, respectively. Raw reads are available at the NCBI SRA under the accession numbers DRR195582 and DRR195583 for MAFF106712 and MAFF302021, respectively.
